# (In)Consistencies in Responses to Sodium Bicarbonate Supplementation: A Randomised, Repeated Measures, Counterbalanced and Double-Blind Study

**DOI:** 10.1371/journal.pone.0143086

**Published:** 2015-11-17

**Authors:** Gabriela Froio de Araujo Dias, Vinicius da Eira Silva, Vitor de Salles Painelli, Craig Sale, Guilherme Giannini Artioli, Bruno Gualano, Bryan Saunders

**Affiliations:** 1 Laboratory of Applied Nutrition and Metabolism, School of Physical Education and Sport, University of Sao Paulo, São Paulo, Brazil; 2 Sport, Health and Performance Enhancement (SHAPE) Research Group, School of Science and Technology, Nottingham Trent University, Nottingham, United Kingdom; Texas A&M University, UNITED STATES

## Abstract

**Objectives:**

Intervention studies do not account for high within-individual variation potentially compromising the magnitude of an effect. Repeat administration of a treatment allows quantification of individual responses and determination of the consistency of responses. We determined the consistency of metabolic and exercise responses following repeated administration of sodium bicarbonate (SB).

**Design and Methods:**

15 physically active males (age 25±4 y; body mass 76.0±7.3 kg; height 1.77±0.05 m) completed six cycling capacity tests at 110% of maximum power output (CCT_110%_) following ingestion of either 0.3 g∙kg^-1^BM of SB (4 trials) or placebo (PL, 2 trials). Blood pH, bicarbonate, base excess and lactate were determined at baseline, pre-exercise, post-exercise and 5-min post-exercise. Total work done (TWD) was recorded as the exercise outcome.

**Results:**

SB supplementation increased blood pH, bicarbonate and base excess prior to every trial (all p ≤ 0.001); absolute changes in pH, bicarbonate and base excess from baseline to pre-exercise were similar in all SB trials (all p > 0.05). Blood lactate was elevated following exercise in all trials (p ≤ 0.001), and was higher in some, but not all, SB trials compared to PL. TWD was not significantly improved with SB vs. PL in any trial (SB1: +3.6%; SB2 +0.3%; SB3: +2.1%; SB4: +6.7%; all p > 0.05), although magnitude-based inferences suggested a 93% likely improvement in SB4. Individual analysis showed ten participants improved in at least one SB trial above the normal variation of the test although five improved in none.

**Conclusions:**

The mechanism for improved exercise with SB was consistently in place prior to exercise, although this only resulted in a likely improvement in one trial. SB does not consistently improve high intensity cycling capacity, with results suggesting that caution should be taken when interpreting the results from single trials as to the efficacy of SB supplementation.

**Trial Registration:**

ClinicalTrials.gov NCT02474628

## Introduction

The effects of sodium bicarbonate (SB) on exercise have been extensively investigated with a meta-analysis indicating that a 0.3 g·kg^-1^BM dose of SB results in a 1.7 ± 2.0% improvement in exercise performance [1]. Nonetheless, evidence remains equivocal, as several studies have reported no effect on exercise theoretically limited by muscle acidosis [2,3,4,5,6,7]. It has recently been suggested that the way in which intervention studies are analysed (*i*.*e*. using mean differences between groups or trials) does not account for the potentially high variation within individuals thus compromising the magnitude of effect of the intervention [8]. However, repeat administration of the experimental treatment in the same set of individuals allows quantification of individual responses and determination of the consistency of these responses [9] in addition to standard analyses.

The relationship between dose and the degree of blood alkalosis following SB ingestion has long been suggested to be weak [10] and the large variability in individual blood pH and bicarbonate responses to supplementation may result in the purported mechanism underlying a potential ergogenic effect of SB not always being present in all individuals which may have contributed to the inconsistent exercise results reported. Saunders et al. [7] recently showed a large variability in the exercise response following SB ingestion. Furthermore, improvements in high-intensity cycling capacity were shown only when individuals experiencing gastrointestinal (GI) discomfort were removed from the analyses, although GI discomfort could not explain a lack of improvement in all individuals. Importantly, the majority of studies have investigated the effect of SB during a solitary trial and not during multiple trials at the same intensity to determine group and individual consistencies in blood and exercise responses.

The aim of this study was to investigate the consistency in blood responses and high-intensity cycling capacity following an identical dose of SB supplementation over repeated trials using the same individuals and exercise protocol. It was hypothesised that SB supplementation would consistently increase blood bicarbonate and pH while subsequent exercise responses would be more variable with individuals not always improving their exercise capacity.

## Materials and Methods

### Participants

**Fig 1:**
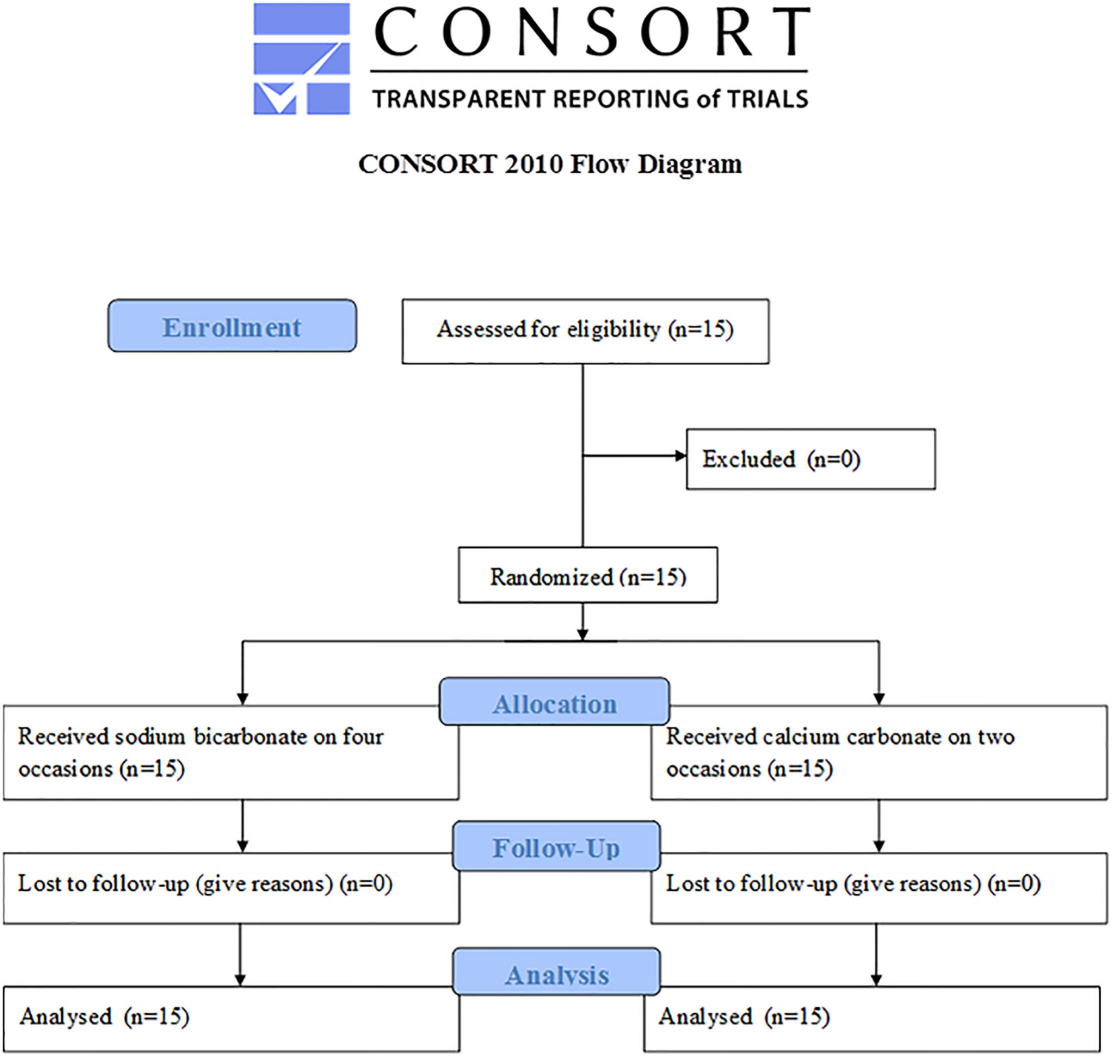
Flow diagram.

Fifteen recreationally active males (mean ± SD; age 25 ± 4 y, body mass 76.0 ± 7.3 kg, height 1.77 ± 0.05 m, maximum cycling output [W_max_] 270 ± 29 W) volunteered and gave their written informed consent to participate in this study. The exclusion criteria included the use of dietary supplements in the past 6 months, the presence of any musculoskeletal disorder, or the use of anabolic steroids. Participant recruitment and data collection was performed between March and September 2014 at the University of São Paulo (Fig 1). The study was first approved by the University of São Paulo's Ethics Review Committee. This study was registered at ClinicalTrials.gov (Identifier: NCT02474628); the study was not registered prior to enrolment of participants as the institution’s ethical committee did not require this as it was not deemed a clinical trial under its descriptors. The protocol for this trial is available as S1 Text and S2 Text. The authors confirm that all ongoing and related trials for this intervention are registered.

### Experimental design

Participants were required to attend the laboratory on eight separate occasions, with all trials being performed at the same time of day to ensure results were not affected by circadian variation [11]. There were two preliminary trials, which comprised of an incremental cycling test to exhaustion to determine W_max_, followed by a familiarisation trial of the high-intensity cycling capacity test to exhaustion at 110% of W_max_ (CCT_110%_). Participants then completed six randomised, repeated measures, counterbalanced and double-blind trials following the ingestion of 0.3 g·kg^-1^BM of either SB on four occasions or calcium carbonate placebo (PL) on two occasions. This was attained through the random drawing of lots containing all fifteen possible trial orders; this process was performed by an individual not involved in any of the data collection. Thereafter, we classified SB1 as the first SB trial performed by participants in their own designation, SB2 as the second SB trial performed by participants and so forth. Similarly, PL1 was classified as the first PL trial performed by participants and PL2 as the second PL trial.

### Design

#### Preliminary testing

Each participant performed a graded cycle capacity test to exhaustion on a cycle ergometer (Lode Excalibur, Germany) to determine W_max_. Individual set up of the cycle ergometer (saddle and handlebar height and length) was determined prior to the W_max_ trial, recorded electronically and maintained for all subsequent trials. Exercise was initiated at 100 W, and increased by 6 W every 15 s (ramp rate of 24 W·min^-1^) until participants reached volitional exhaustion. The last completed stage plus the fraction of time spent in the final non-completed stage multiplied by 6 W was defined as an individual’s W_max_.

All participants performed a familiarisation of the CCT_110%_ to minimise any learning effect during the main trials. A 5 min cycling warm up was performed at 100 W followed by a 3 min period of self-selected stretching. Due to the intense nature of the exercise, each participant’s CCT_110%_ was incremented over the first 30 s, which corresponded to 80% W_max_ during the first 15 s, 95% W_max_ over the second 15 s followed by 110% W_max_ until volitional exhaustion. Participants pedalled at a self-selected pedal cadence (range 70–110 rev·min^-1^ across participants) and were required to maintain this cadence throughout the entire test. Verbal encouragement was given throughout. Volitional exhaustion was deemed to have occurred when participants dropped below 60 rev·min^-1^ for over 3 consecutive seconds, at which point they were instructed to stop pedalling. TWD (kJ) was recorded as the outcome measures for all tests. The CCT_110%_ is reliable within recreationally active individuals following a single familiarisation session; the coefficient of variation (CV) for TWD has been shown to be 4.94% [12].

### Main trials

Twenty-four hours prior to the main trials, participants were required to refrain from alcohol, caffeine and any strenuous exercise. Food intake was monitored during the twenty-four hours prior to the first main trial using a food diary and replicated prior to the remaining main trials. Upon arrival at the laboratory ~2 h before the CCT_110%_, baseline finger-prick and venous blood samples were taken from all participants. They were then required to ingest 0.3 g·kg^-1^BM of SB or matching PL (calcium carbonate) alongside 500 ml water within a 10-minute period. All supplements were administered in unmarked gelatine capsules identical in size, colour and overall appearance, and participants were supervised to ensure 100% compliance. Exercise commenced 90 minutes following the ingestion of supplements; participants performed the CCT_110%_ as described above for the familiarisation trial.

Finger-prick blood samples were taken at baseline, immediately pre-exercise, immediately post-exercise and 5-min post-exercise and analysed for lactate concentration (Accutrend Lactate, Roche Diagnostics, Switzerland). Venous blood samples were taken at identical time points from the antecubital vein and analysed for blood pH, bicarbonate and base excess (Rapid Point 350, Siemens, Germany). The baseline, post-exercise and 5-min post-exercise CVs for pH, bicarbonate and base excess ranged from 0.07 ± 0.03% to 2.77 ± 2.2%. All blood samples were taken with the individuals in a seated and upright position. Participants rated their intensity of stomachache, sickness and headache on a ten point scale [13] immediately prior to ingestion of the supplement, immediately prior to exercise and immediately post-exercise. Participants were allowed to drink water *ad libitum* throughout.

### Statistical analysis

All data were analysed using the SAS statistical package, (SAS 9.2, SAS Institute Inc., USA) and are presented as mean ± 1SD. Blood data (absolute and deltas) were analysed using a mixed model ANOVA with repeated measures (supplement x trial x time) to determine any differences in blood pH, bicarbonate, base excess and lactate. Tukey tests were used for *post-hoc* analyses to determine where any differences lay as indicated by the ANOVA. Exercise capacity data were analysed using a two-way ANOVA (supplement x trial) with repeated measures to determine any differences. Further analyses were performed to mimic an alternative design in which each SB trial had been performed as an independent study to highlight the variability and inconsistency in exercise responses. Since no differences in exercise capacity were seen between trials for PL, data from the two trials were pooled and the mean used as the PL exercise capacity value in order to perform these further individual analyses. Thereafter, t-tests were performed between all trials for TWD to mimic the results if each SB trial been an independent study. Statistical significance was accepted at p ≤ 0.05. In addition, magnitude based inferences [14] were used to determine the practical significance of SB on the CCT_110%_ using a spreadsheet to establish the likelihood of a meaningful effect on exercise capacity. The smallest worthwhile improvement in TWD was 1.27 kJ, which was equivalent to half the unbiased typical error associated with the measurement. Qualitative descriptors were assigned to the positive percentile scores as follows: <1%, *almost certainly not*; 1–5%, *very unlikely*; 5–25%, *unlikely*; 25–75%, *possibly*; 75–95%, *likely*; 95–99%, *very likely*; >99%, *almost certainly* [15].

## Results

### Blood responses

**Fig 2:**
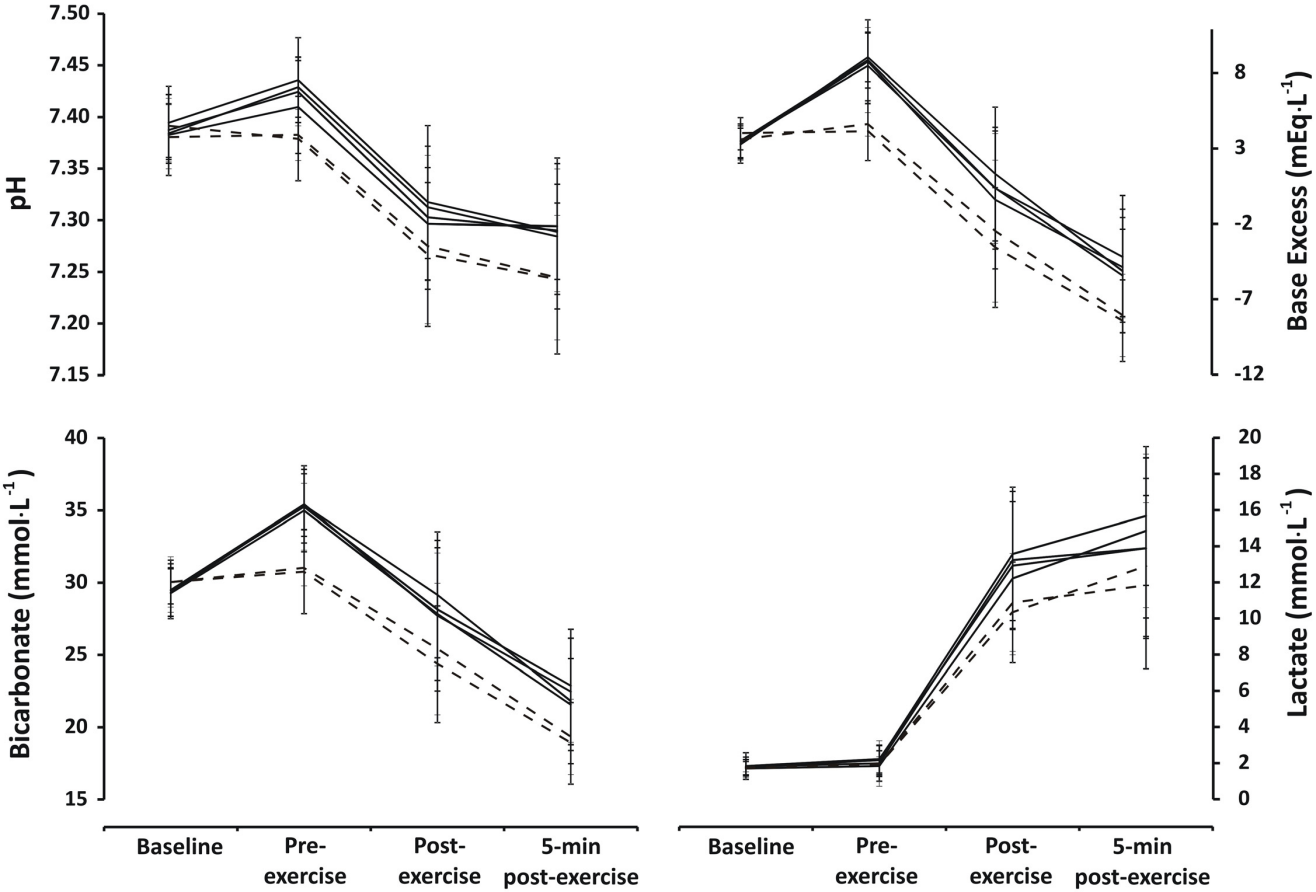
Line graphs for blood measurements (mean ± 1SD) at Baseline, Pre-exercise, Post-exercise and 5-min post-exercise. Panel A displays pH; Panel B displays bicarbonate; Panel C displays base excess; Panel D displays lactate. PL trials are represented by dashed lines and SB trials are represented by solid lines.

There was no effect of trial order on any blood parameter (all p > 0.05). Baseline blood pH, bicarbonate, base excess and lactate were similar between all trials (Fig 2). There were significant increases in blood pH, bicarbonate and base excess between baseline and pre-exercise in all trials where SB was consumed (supplement x trial x time interaction p ≤ 0.001, *post hoc* all p ≤ 0.05; see S1 Table), but no significant alterations were shown in PL1 or PL2 (Fig 2). Blood lactate was not significantly different between trials at baseline or pre-exercise (supplement x trial x time interaction p = 0.21).

Immediately post-exercise and 5-min post-exercise pH, bicarbonate and base excess values were significantly reduced from immediately pre-exercise (time effect p ≤ 0.001; see S1 Table). The post-exercise blood pH values in SB1 and SB4 were higher than both PL trials, while bicarbonate and base excess at the same time point were higher in all SB trials compared to both PL trials (all p ≤ 0.05). Exercise significantly increased lactate in all trials (main time effect p < 0.0001), with increased post-exercise values shown in all SB versus PL trials except SB2.

The changes from baseline to pre-exercise in blood pH, bicarbonate and base excess during all SB trials were different from both PL trials (all p ≤ 0.05) with no differences between SB trials (all p > 0.05; Table 1). There were no differences between any trials in the change in lactate from baseline to pre-exercise (all p > 0.05). The change in pH from pre-exercise to post-exercise was different in all SB trials compared to PL1 (all p ≤ 0.05), but only in SB2 compared to PL2 (Table 1). There were no differences between any trials in the change in bicarbonate and base excess from pre-exercise to post-exercise (all p > 0.05). Pre-exercise to post-exercise change in lactate in SB1 and SB4 was different from PL2 with a trend towards a difference from PL1, while there was also a trend towards a difference between SB3 and PL2 (Table 1).

**Table 1:** Changes in blood pH, bicarbonate, base excess and lactate from Baseline to Pre-exercise and Pre-exercise to Post-exercise. ^#^Different from PL1 at the same time point (p ≤ 0.05). ^^^Different from PL2 at the same time point (p ≤ 0.05). ^£^Trend towards a difference from PL1 (p ≤ 0.08). ^$^Trend towards a difference from PL2 (p ≤ 0.08).

	Δ Baseline to Pre-exercise	Δ Pre-exercise to Post-exercise
**pH**		
PL 1	+0.002±0.031	-0.107±0.070
PL 2	-0.012±0.045	-0.112±0.054
SB 1	+0.045±0.029^#^^	-0.116±0.077^#^
SB 2	+0.037±0.043^#^^	-0.122±0.053^#^^
SB 3	+0.027±0.054^#^^	-0.113±0.050^#^
SB 4	+0.041±0.031^#^^	-0.118±0.052^#^
**Bicarbonate (mmol·L** ^**-1**^ **)**		
PL 1	+1.0±1.6	-5.6±4.0
PL 2	+0.7±2.7	-6.4±4.8
SB 1	+6.1±2.3^#^^	-6.2±4.5
SB 2	+5.8±1.6^#^^	-7.1±3.6
SB 3	+5.9±2.7^#^^	-7.7±3.3
SB 4	+5.7±2.9^#^^	-7.2±4.1
**Base excess (mEq·L** ^**-1**^ **)**		
PL 1	+1.0±1.0	-7.1±4.4
PL 2	+0.2±1.7	-7.8±4.4
SB 1	+5.8±1.9^#^^	-7.7±4.4
SB 2	+5.0±2.5^#^^	-8.1±4.5
SB 3	+5.4±2.6^#^^	-9.2±3.1
SB 4	+5.4±2.1^#^^	-8.5±3.9
**Lactate (mmol·L** ^**-1**^ **)**		
PL 1	+0.1±1.4	+8.9±2.5
PL 2	+0.3±0.8	+8.4±2.8
SB 1	+0.2±1.2	+11.3±4.3^^£^
SB 2	+0.1±0.6	+10.4±4.5
SB 3	+0.4±0.9	+10.7±3.6^$^
SB 4	+0.4±0.7	+11.4±4.0^^£^

Individual analysis of baseline to pre-exercise changes in blood measures showed that SB failed to increase blood pH on a total of only ten out of sixty occasions; this occurred in two separate trials for two individuals and one single trial for a further six. SB increased blood bicarbonate on all occasions in all individuals except one who failed to increase circulating bicarbonate prior to their final SB trial. Base excess was increased following SB supplementation on all but two occasions with two individuals showing no increases in a single SB trial.

### Exercise responses

There was no effect of trial order on TWD (p = 0.90), indicating there was no learning effect over time. Mixed model ANOVA showed no effect of supplementation (p = 0.83) or trial (p = 0.25) on TWD, although there was a significant interaction effect (supplement x trial, p = 0.04); *post hoc* analyses revealed no differences between trials (all p > 0.05) except between SB2 and SB4 (p = 0.02). The CV for PL trials was 6.2 ± 5.0% (range: 0.5 to 13.2%), while the CV for the SB trials was 7.4 ± 3.2 (range: 2.5 to 14.8%).

**Fig 3:**
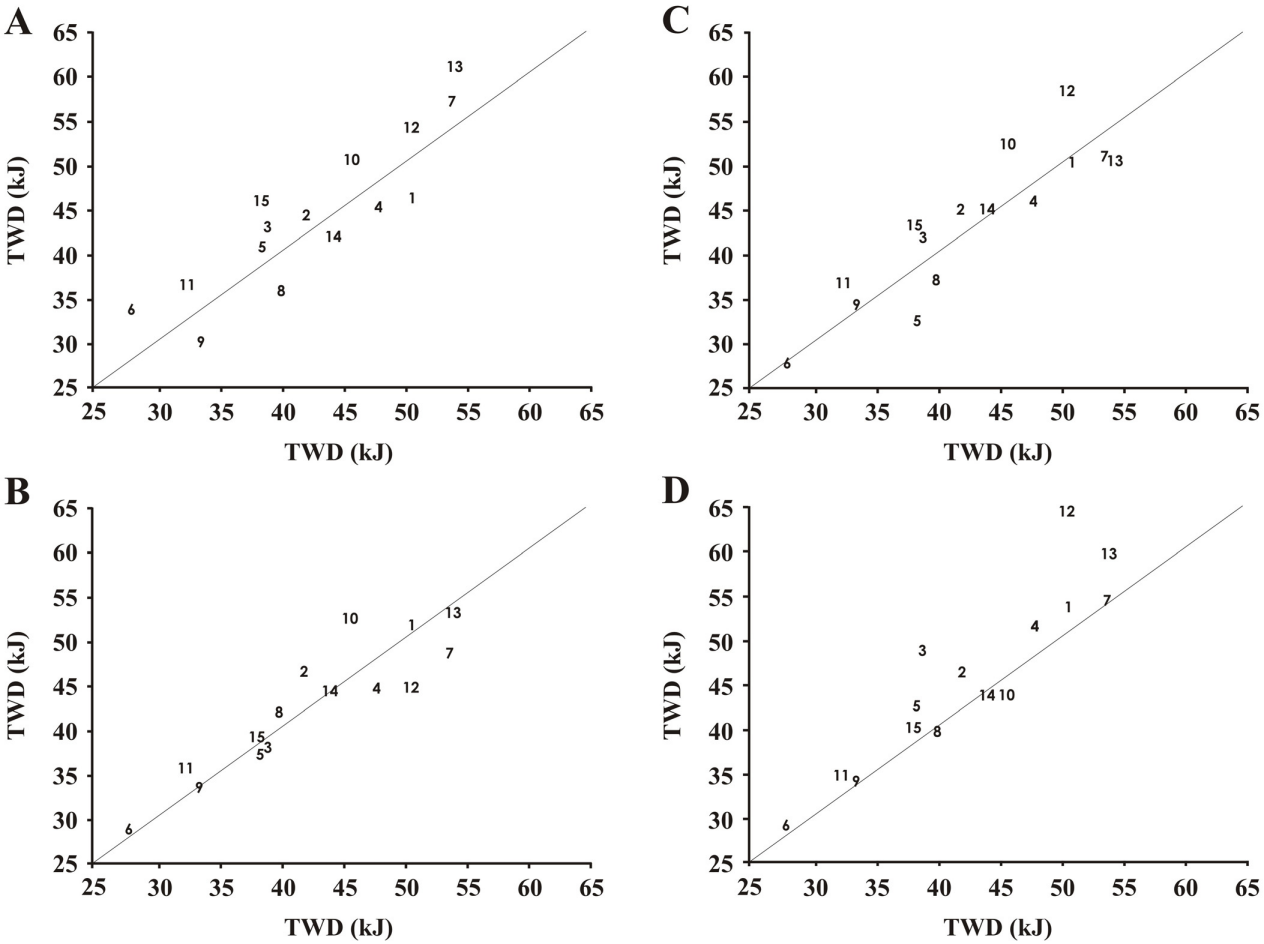
Scatterplots of all SB trials (y-axes) versus PL (x-axes) for all participants (1–15). Panel A displays SB1; Panel B displays SB2; Panel C displays SB3; Panel D displays SB4. Individuals 1 and 9 are those who reported substantial side effects in SB1 and participant 5 reported GI disturbance in SB3.

Since ANOVA, t-tests and MBIs indicated no difference between PL1 and PL2 (p = 0.39, p = 0.2, *unclear)*, data were combined and the mean used as the PL trial and further comparisons were performed according to these data (SB trials *versus* PL). Mixed model ANOVA showed SB did not improve exercise capacity compared to PL in any trial (all p > 0.05; Table 2), although MBIs indicated a *likely* improvement during SB4 and a *possible* improvement in SB1 and SB3 (Table 2). Furthermore, t-tests showed that the difference between PL and SB4 was significant (p = 0.01; Table 2). There was a large degree of individual variability in exercise capacity between PL and all SB trials with a difference in TWD ranging from -6.1 to +13.4 kJ. Individual analysis was performed by classifying the difference between each SB trial and PL using the CV from the PL trials from these participants which corresponded to 6.2%. Exercise capacity was considered improved when the difference from PL was in excess of +6.2%; unaffected when this difference was between -6.2% and +6.2% and reduced when the difference exceeded -6.2%. This individual analysis revealed ten participants improved in at least one SB trial above the normal variation of the test, while five did not improve in a single trial. Only one participant improved in all four trials; three improved in three trials; three improved in two trials and three improved in one trial (Fig 3). A total of 22 out of the 60 trials with SB were improved above the CV for these individuals, while 27 were within the variation and 11 were below.

**Table 2:** Mean ± 1SD percentage differences from PL; 95% confidence intervals for mean difference; t-test p values; number of individuals above, within and below the CV versus PL; and practical significant of effects during all SB trials.

	Mean difference from PL (%)	95% CI(lower to upper)	T-test	Above/Within/Below	Chances of substantial improvement	
					%	Qualitative
**SB1**	+3.6 ± 10.2	-2.1 to +9.3	0.21	6/4/5	54	possibly
**SB2**	+0.3 ± 7.2	-3.6 to +4.3	0.94	3/9/3	7	unlikely
**SB3**	+2.1 ± 9.1	-3.0 to +7.1	0.45	6/6/3	32	possibly
**SB4**	+6.7 ± 8.8	+1.8 to +11.5	0.01	7/8/0	93	likely

There was no effect of supplement, trial or time on intensity of stomach ache (all p > 0.05); there was an effect of time on intensity of headache (p = 0.02) although there was no effect of supplement, trial or any interaction effects (all p > 0.05). There was an effect of time (p = 0.0002) and trend towards an effect of supplement (p = 0.09) and trial (p = 0.06) on sickness; *post-hoc* analysis revealed pre-exercise values in SB1 and SB3 to be significantly elevated above pre-exercise values in all other trials (p ≤ 0.05). A total of three reports of stomachache (range: 1–2) were reported immediately prior to exercise during the SB trials, while incidence of headache (range: 1–4) was reported on four occasions. Analysis revealed between two and seven participants per trial reported symptoms of sickness immediately prior to exercise following SB; sickness was reported on a total of 21 out of the 60 occasions following SB supplementation. On three occasions was an individual’s score above 6; this occurred for intensity of sickness in two individuals prior to SB1 and one prior to SB3. When these individuals were removed from the analyses, TWD in SB1 was improved versus PL (N = 13; 45.2 ± 8.3 vs. 43.0 ± 7.9 kJ; +5.7 ± 9.3%; p = 0.05; MBI: 81% *likely*) though not in SB3 (N = 14; 44.5 ± 8.2 vs. 43.3 ± 8.2 kJ; +3.3 ± 8.0%; p = 0.21; MBI: 50% *possibly*).

## Discussion

This is the first study to show that blood responses following repeat administration of an identical dose of SB in the same population results in near identical increases in pH, bicarbonate and base excess, although this only translated into an improved exercise capacity in one of the trials in recreationally active individuals. There were a number of non-responders as five individuals did not improve exercise capacity above the variation of the test in any trial, although the majority of individuals improved in some trials with SB and one improved in all. SB results in highly consistent blood responses in the same individuals, although this does not necessarily translate into an improved exercise capacity.

SB ingestion resulted in alkalosis prior to exercise in all participants; mean increases in blood bicarbonate of between +5.7 and +6.1 mmol∙L^–1^ were similar in all trials and higher than the average increases (+3.9 ± 0.9 mmol∙L^–1^) in the literature as shown by Carr et al. [1] in a meta-analysis. SB only failed to increase blood bicarbonate on one occasion in all trials and resultant increases in blood pH and base excess were also consistent in all SB trials. Therefore, pre-exercise blood parameters cannot explain a lack of an ergogenic effect, since these were always increased prior to exercise, suggesting the mechanism for an improved exercise capacity was in place prior to every trial. Since the absolute increases in blood pH, bicarbonate and base excess prior to exercise were extremely similar between all SB trials, it may be, therefore, that an individual cannot always make full use of this blood alkalosis and enhanced buffering capacity. We speculate that this may be due to a variability in monocarboxylate (MCT) transporter protein activity following SB supplementation and the rate at which H^+^ is removed from the working cell. Future research should establish the activity profile of MCT1 and MCT4 following SB ingestion and the consistency thereof.

Repeated-bout high-intensity activity has been shown to be highly susceptible to improvements with SB [16, 17], with evidence to suggest that multiple bouts of supra-maximal exercise results in increased muscle acidosis compared to continuous supra-maximal exercise [18, 19]. Nonetheless, the CCT_110%_ has been suggested to be a highly sensitive test to increases in buffering capacity since similar improvements were consistently shown following increases in muscle buffering capacity achieved via β-alanine supplementation in recreationally active participants [20, 21, 22]; importantly, exercise improvements in these studies were shown in almost all individuals. Despite this, CCT_110%_ was only improved in one out of four trials following SB supplementation. Individual analysis revealed that five individuals did not improve exercise capacity in a single trial following SB supplementation. This is the first study to suggest some individuals may be consistent non-responders to SB although all participants were improved in at least one trial when improvements above the CV were disregarded. Additionally, only one individual in our study improved above the variation in every trial with SB, while the majority of the remaining individuals improved in some, but not all, trials. The results of this study highlight the difficulty in interpreting results from a solitary trial following SB since it appears that almost no individual consistently improves following supplementation despite prior alkalosis.

In this study we performed standard statistical analyses to determine the effect of supplementation on exercise capacity and the mixed model ANOVA with repeated measures showed no effect of SB compared to PL. Thus, additional analyses were performed to investigate group and individual responses during the repeated trials. The various data analyses techniques performed in this study further support the difficulty in reporting on the results from a single trial, since ANOVA revealed no effect during any trial. However, if only a solitary SB trial had been performed and routine analyses employed (t-tests), then SB1, SB2 and SB3 would not have resulted in a beneficial effect while SB4 would be in support of an ergogenic effect. Additionally, magnitude based inferences support the results of the independent t-test with respect to SB4. These data support the claims of Hecksteden et al. [8] that variability in responses may compromise the magnitude of effect of an intervention. Additional analyses suggest that some individuals are consistent non-responders to SB while others respond positively only occasionally, further highlighting the difficulty in interpreting only grouped means from a single trial since subsequent exercise responses following SB supplementation appear to be inconsistent. The present study provides data to support the necessity in employing multiple trials to determine the effect of SB supplementation on exercise due to the variability and inconsistency in exercise responses. Furthermore, supplementing conventional statistics and analysing both group and individual responses appear necessary in order to draw appropriate conclusions from subsequent results.

Similar to Saunders et al. [7], the associated side effects of SB supplementation appeared to affect exercise responses in at least one trial (SB1). A threshold of ≥6 in any individual score was considered sufficient to influence exercise, and data were subsequently reanalysed following the exclusion of these PL and SB results. This resulted in a significant improvement in exercise capacity in SB1, which was previously not improved. The results give further support to the notion that associated side effects may cause SB supplementation to be ergolytic despite resulting in pre-exercise alkalosis. However, associated side effects appear to be inconsistent with discomfort reported across all trials. This means that trialling SB during training will likely not increase an individual’s resistance to associated side effects. Therefore, a more chronic supplementation protocol may be merited to avoid the possibility of these side-effects hindering performance; supplementation over a period of several days has been shown to result in increased blood parameters even 48 hours after cessation [23], meaning acute supplementation immediately prior to competition is unnecessary.

The CCT_110%_ has previously been shown to have a CV of ~5% for TWD using recreationally active males [12], indicating it to be a reliable exercise capacity test for untrained individuals. The CV between placebo trials in this study was slightly higher at 6.2%. However, this is likely due to the different study aims; this study investigated the ergogenic effect of a supplement, which may have contributed to an increased expectancy [24]. The CV for SB trials was 7.4% across all individuals; this is higher than the 2.1% shown with 2000 m rowing in well-trained rowers [25]. The discrepancy between these two studies may be due to the exercise protocol employed or the different sample populations [26]. Although performance was not improved with SB in the study by Carr et al. [25], trained individuals may be better able to consistently take advantage of an increased buffering capacity; the present study appears to indicate that recreationally active individuals are unable to consistently take advantage of an increased extracellular buffering capacity. Future research should investigate the effect of repeated SB trials with athletes who are likely to exhibit a lower CV for a chosen test.

In conclusion, the mechanism for improved exercise with SB was consistently in place prior to every trial, although this only translated into a likely improved exercise capacity on one occasion. These results suggest that SB may not always improve exercise in the same individual, meaning caution should be taken when interpreting the results from a single trial. In light of these data, practitioners should trial SB on multiple occasions in order to categorise individuals into non-responders and potential responders.

## Supporting Information

S1 CONSORT Checklist CONSORT Checklist. (DOC)

S1 Data Data file. (XLS)

S1 Table pH, lactate, bicarbonate and base excess at Baseline, Pre-exercise, Post-exercise and 5-min post-exercise in all trials. (DOC)

S1 Text Ethics application Portuguese. (DOCX)

S2 Text Ethics application English. (DOCX)
