# Supplementary material for: (In)Consistencies in Responses to Sodium Bicarbonate Supplementation: A Randomised, Repeated Measures, Counterbalanced and Double-Blind Study
Source: PLoS One. 2015 Nov 17;10(11):e0143086. doi: 10.1371/journal.pone.0143086 (PMC4648485; doi:10.1371/journal.pone.0143086)
Supplement: S1 Table — (DOC) [file pone.0143086.s003.doc]

Supplemental Digital Content 1**.[[1]](#footnote-2)**

|  | **Baseline** | **Pre-exercise** | **Post-exercise** | **5-min post-exercise** |
| --- | --- | --- | --- | --- |
|  |  |  |  |  |
| **pH** |  |  |  |  |
| PL1 | 7.381±0.031 | 7.383±0.025 | 7.275±0.075^ | 7.244±0.060^* |
| PL2 | 7.391±0.030 | 7.379±0.041 | 7.267±0.070^ | 7.244±0.073^*^ |
| SB1 | 7.384±0.029 | 7.429±0.029^* | 7.313±0.079^* | 7.284±0.070^* |
| SB2 | 7.387±0.031 | 7.424±0.033^* | 7.303±0.060^ | 7.290±0.059^*^ |
| SB3 | 7.383±0.039 | 7.410±0.045^ | 7.297±0.055^ | 7.294±0.066^*^ |
| SB4 | 7.401±0.038 | 7.436±0.041^* | 7.317±0.054^* | 7.289±0.046^* |
|  |  |  |  |  |
| **Bicarbonate (mmol·L^-1^)** | |  |  |  |
| PL1 | 30.1±1.7 | 31.0±1.2 | 25.4±4.5^ | 19.3±2.6^ |
| PL2 | 30.0±1.5 | 30.8±2.9 | 24.4±4.0^ | 18.9±2.8^ |
| SB1 | 29.3±1.6 | 35.4±2.2^* | 29.2±4.4^* | 21.8±4.3^* |
| SB2 | 29.5±1.5 | 35.2±1.6^* | 28.1±3.9^* | 22.9±3.9^* |
| SB3 | 29.5±1.8 | 35.4±2.7^* | 27.7±5.2^* | 22.5±3.7^* |
| SB4 | 29.3±1.8 | 35.0±2.8^* | 27.8±4.6^* | 21.6±3.2^* |
|  |  |  |  |  |
| **Base excess (mmol·L^-1^)** | |  |  |  |
| PL1 | 3.6±1.4 | 4.6±0.8 | -2.5±4.7^ | -8.1±2.7^ |
| PL2 | 4.0±1.1 | 4.2±2.0 | -3.6±4.0^ | -8.4±2.7^ |
| SB1 | 3.3±1.2 | 9.0±1.6^* | 1.3±4.4^* | -5.1±4.1^* |
| SB2 | 3.5±1.2 | 8.5±2.5^* | 0.4±3.6^* | -4.2±4.1^* |
| SB3 | 3.3±1.0 | 8.8±2.8^* | -0.4±4.6^* | -4.9±3.3^* |
| SB4 | 3.5±1.1 | 8.9±1.8^* | 0.4±4.0^* | -5.4±3.1^* |
|  |  |  |  |  |
| **Lactate (mmol·L^-1^)** | |  |  |  |
| PL1 | 1.8±0.3 | 2.0±1.3 | 10.9±2.7^ | 11.8±4.6 |
| PL2 | 1.7±0.4 | 2.0±0.7 | 10.3±2.8^ | 12.9±3.9^ |
| SB1 | 1.8±0.5 | 2.0±1.0 | 13.2±3.8^* | 13.9±5.0 |
| SB2 | 1.7±0.5 | 1.8±0.5 | 12.2±4.2^ | 14.8±4.2^* |
| SB3 | 1.8±0.7 | 2.2±0.8 | 12.9±3.5^* | 13.9±3.9 |
| SB4 | 1.8±0.4 | 2.2±0.8 | 13.6±3.7^* | 15.7±3.8^* |

1. Supplemental Digital Content 1. pH, lactate, bicarbonate and base excess (mean ± 1SD) at Baseline, Pre-exercise, Post-exercise and 5-min post-exercise in all trials. ^denoting a significant post-hoc difference from previous time point (*P* < 0.05). ^*^denoting a significant post-hoc difference from PL1 and PL2 at the same timepoint (*P* < 0.05). [↑](#footnote-ref-2)
