# Supplementary material for: (In)Consistencies in Responses to Sodium Bicarbonate Supplementation: A Randomised, Repeated Measures, Counterbalanced and Double-Blind Study
Source: PLoS One. 2015 Nov 17;10(11):e0143086. doi: 10.1371/journal.pone.0143086 (PMC4648485; doi:10.1371/journal.pone.0143086)
Supplement: S1 Text — (DOCX) [file pone.0143086.s004.docx]

UNIVERSIDADE DE SÃO PAULO

ESCOLA DE EDUCAÇÃO FÍSICA E ESPORTE

LABORATÓRIO DE NUTRIÇÃO E METABOLISMO APLICADO À ATIVIDADE MOTORA

Efeitos da suplementação suplementação de bicarbonato de sódio sobre a capacidade física em múltiplos testes.

Pesquisador Gerente: Bryan Saunders

Pesquisador Responsável: Prof. Dr. Bruno Gualano

DEPARTAMENTO DE BIODINÂMICA DO MOVIMENTO DO CORPO HUMANO

2013

**Resumo**

Diversos estudos têm demonstrado que a acidose muscular, ocasionada pelo acúmulo de íons H^+^ no interior da célula muscular, é um fator limitante para o desempenho físico durante exercícios físicos de alta intensidade. Assim, estratégias com o objetivo de atenuar a queda do pH intramuscular têm o potencial de se destacar no cenário esportivo. Dentre elas, a suplementação de bicarbonato de sódio vem recebendo especial atenção em anos recentes. A suplementação com este composto pode aumentar os níveis sanguíneos de pH e bicarbonato, aumentando a capacidade de tamponamento extracelular, e por consequência, o desempenho físico. Apesar dos seus comprovados efeitos metabólicos, a eficácia ergogênica da suplementação de bicarbonato de sódio ainda permanece controversa. As inconsistências nos resultados das pesquisas investigando a eficácia ergogênica da suplementação com bicarbonato de sódio podem ser parcialmente atribuídas à: administração de diferentes doses do suplemento, modelos de exercício que não são limitados pela acidose intramuscular, variação individual da resposta à suplementação e desconforto gastrointestinal acometendo alguns indivíduos. Em concordância com estas suposições, nosso grupo recentemente verificou que quando os participantes que sofreram desconforto gastrointestinal foram removidos da análise, a suplementação de bicarbonato de sódio melhorou em 5% o trabalho total realizado a 110% potência máxima. Além disso, foi constatado que a suplementação de bicarbonato de sódio não foi eficaz em todos os indivíduos, inclusive em participantes com respostas positivas à suplementação.

Assim, o objetivo do presente estudo é determinar se há consistência do efeito ergogênico do bicarbonato de sódio em indivíduos que melhoram a capacidade física, e verificar se a ausência de resposta também é consistente entre os indivíduos cuja capacidade física não se alterarou. Para tanto, serão recrutados aproximadamente 20 homens fisicamente ativos. Esta pesquisa terá um desenho randomizado, controlado por placebo, duplo-cego cross-over, o qual os sujeitos serão alocados em 2 tratamentos diferentes: Placebo e Bicarbonato de Sódio (300mg/kg). Para cada um dos tratamentos neste segundo estudo, os indivíduos serão submetidos a testes de capacidade física para verificação de alterações neste parâmetro em virtude dos tratamentos. Antes de cada visita experimental será padronizada uma dieta aos voluntários. Serão analisadas variáveis descritivas (média, mediana e desvio-padrão), e análise da qualidade dos dados (verificar se a distribuição é normal e se a variância é homogênea).

**Introdução**

A fadiga muscular é um fenômeno estudado há décadas e é caracterizado pela incapacidade do músculo esquelético em manter uma determinada tensão ou de manter o exercício físico a uma dada intensidade (Sahlin, 1992). Até hoje as causas para a sua ocorrência permanecem incompreendidas. Apesar disso, evidências indicam papel particularmente relevante de alguns eventos no surgimento da fadiga, tais como a inibição de enzimas que participam da transferência de energia, a diminuição da sensibilidade aos íons cálcio (Ca^2+^) no sítio da troponina, a diminuição da liberação ou da re-captação de Ca^2+^ no retículo sarcoplasmático e a depleção de substratos energéticos (Sahlin, 1992; Allen et al., 2008).

Apesar disso, no que diz respeito aos exercícios de alta intensidade, algumas causas têm ganhado destaque na literatura. Dentre elas, o acúmulo de alguns metabólitos no interior da célula muscular, em específico, de íons H^+^, gerando uma queda do pH muscular, isto é, uma acidose intramuscular. Nesse sentido, estudos já demonstraram que os íons H^+^ possuem a capacidade de competir com os íons Ca^2+^ pelo sítio de ligação da troponina, prejudicando a capacidade da maquinaria contrátil de operar efetivamente (Donaldson et al., 1978; Fabiato & Fabiato, 1978). Além disso, a queda do pH muscular causada pelos íons H^+^ pode levar à inibição da ressíntese de fosforilcreatina (Harris et al., 1976) e à inibição de enzimas importantes da via glicolítica (Sutton et al., 1981), limitando o processo de produção de energia para a contração muscular. Diante dessa importância da regulação do pH durante o exercício de alta intensidade, estratégias que contribuam para a manutenção do equilíbrio ácido-base tornam-se potencialmente ergogênicas.

Nesse sentido, a suplementação de bicarbonato de sódio vem recebendo especial destaque na literatura. Diversos estudos têm demonstrado que a suplementação de bicarbonato de sódio eleva significantemente o pH sanguíneo e as concentrações de bicarbonato no sangue (Requena et al., 2005). Tais alterações aumentam o fluxo de íons H^+^ e lactato para fora do músculo ativo. Isso ocorre devido a um aumento da atividade do co-transportador lactato/H^+^ chamado monocarboxilase (MCT, do inglês *monocarboxylase transporter*), o qual se torna mais ativo conforme o gradiente intracelular/extracelular de íons H^+^ aumenta (Mainwood & Worsley-Brown, 1975; Mainwood & Cechetto, 1980). Com isso, o pH intramuscular diminui mais lentamente, havendo menor interferência da acidose sobre o processo contrátil e produção de ATP pela via glicolítica, e portanto, atrasando o início da fadiga.

Apesar dos seus comprovados efeitos metabólicos, a eficácia ergogênica da suplementação de bicarbonato de sódio ainda permanece controversa (Price & Simons, 2010). As inconsistências nos resultados das pesquisas investigando a eficácia ergogênica da suplementação com bicarbonato de sódio podem ser parcialmente atribuidas à: administração de diferentes doses do suplemento (Horswill et al., 1988), modelos de exercício que não são limitados pela acidose intramuscular (Linderman et al., 1992), variação individual da resposta à suplementação (Price & Simons, 2010) e desconforto gastrointestinal acometendo alguns indivíduos (McNaughton, 1992). Em concordância com estas suposições, Saunders et al. (in press) verificaram que quando os participantes que sofreram desconforto gastrointestinal foram removidos da análise, a suplementação de bicarbonato de sódio melhorou em 5% o trabalho total realizado a 110% potência máxima. Além disso, os autores constataram que a suplementação de bicarbonato de sódio não foi eficaz em todos os indivíduos, inclusive em participantes com respostas positivas à suplementação.

**Hipótese -** A performance e a resposta sanguínea em testes múltiplos não serão consistentes com a suplementação de bicarbonato de sódio no mesmo indivíduo.

**Objetivos**

***Objetivo geral:*** Investigar o efeito de bicarbonato de sódio sobre a capacidade física durante testes consecutivos.

***Objetivo específico:*** Determinar se há consistência do efeito ergogênico do bicarbonato de sódio em indivíduos que melhoram a capacidade física, e verificar se a ausência de resposta também é consistente entre os indivíduos cuja capacidade física não se alterarou.

**Metodologia**

*Seleção da Amostra*

Serão selecionados 20 indivíduos do sexo masculino, de 18 a 30 anos, para participar da pesquisa. Após explicação detalhada sobre a pesquisa, riscos e benefícios envolvidos, todos os participantes deverão assinar o termo de consentimento livre e esclarecido. Para serem incluídos neste estudo, os participantes deverão ser 1) saudáveis, sem qualquer doença cardiovascular ou acometimento do aparelho locomotor; 2) fisicamente ativos, isto é, praticantes de atividade física, porém, sem estarem engajados em qualquer programa regular de treino e/ou competição. Serão excluídos do estudo os participantes que estejam fazendo uso ou tenham feito uso de creatina e beta-alanina nos últimos 3 e 6 meses, respectivamente.

*Local e Duração do Estudo*

As análises sanguíneas serão conduzidas no LABNUTRI (Laboratório de Nutrição e Metabolismo Aplicados à Atividade Motora) da EEFE-USP (Escola de Educação Física e Esporte da Universidade de São Paulo). Os testes físicos serão conduzidos no LADESP (Laboratório de Determinantes Energéticos do Desempenho Esportivo) da EEFE-USP.

O estudo terá duração aproximada de 2 semestres.

*Desenho Experimental*

O protocolo consistirá de nove visitas ao laboratório, que será mantido sempre em 19,0 ºC, com umidade relativa de aproximadamente 64%.

Em ordem, a primeira visita consistirá de um teste de potência máxima. A segunda visita consistirá de uma familiarização ao teste físico. As sessões subsequentes serão as sessões experimentais, onde os voluntários serão submetidos a dois tratamentos diferentes, os quais serão separados por períodos de *washout* de pelo menos 48 horas: Suplementação de bicarbonato de sódio (300 miligramas por quilograma de peso corporal) e suplementação de placebo (carbonato de cálcio, na mesma dosagem de bicarbonato de sódio).

As sessões experimentais serão conduzidas com um desenho duplo-cego, *crossover* contrabalanceado, controlado por placebo. Durante as sessões experimentais, serão realizadas coletas sanguíneas para a avaliação do pH, lactato e bicarbonato sanguíneos antes da ingestão dos suplementos, 90 minutos após a ingestão dos mesmos e imediatamente após o teste físico. Todos os testes físicos serão realizados no mesmo equipamento (Lode, Netherlands), onde os voluntários serão testados para o tempo até a exaustão e para o trabalho total.

As sessões experimentais serão conduzidas no período da tarde. A fim de minimizar as diferenças nas concentrações iniciais de glicogênio muscular, nosso grupo padronizará o café da manhã e almoço que precede cada um dos testes experimentais (75,6 ± 4,5 kJ/kg, contendo 41 ± 1 % carboidrato, 19 ± 1 % proteína, e 40 ± 1 % gordura). Os participantes serão orientados a manter um recordatório alimentar das últimas 24 horas antes de cada teste. Eles também serão instruídos a não realizar nenhum tipo de atividade física no dia anterior a cada teste. Eles realizarão o café da manhã padronizado às 09:00hs da manhã, o almoço às 12:00hs e se apresentarão ao laboratório para os testes físicos e coletas sanguíneas às 14:00hs da tarde. Todos os testes e coletas serão realizados no mesmo horário.

*Protocolo de Suplementação*

Será adotado um protocolo de suplementação aguda de bicarbonato de sódio e placebo. A dose de bicarbonato de sódio utilizada será de 300 miligramas por quilograma de peso corporal de forma a tentar mimetizar as doses utilizadas em estudos anteriores que verificaram um efeito ergogênico desta estratégia nutricional (Artioli et al., 2007; Painelli et al., 2013). O suplemento será administrado em cápsulas gelatinosas de mesmo tamanho e cor. O placebo utilizado será o carbonato de cálcio. Para a manutenção do desenho duplo-cego, ele será administrado em cápsulas de mesmo tamanho e cor das cápsulas de bicarbonato de sódio. Além disso, o placebo será administrado em uma quantidade de cápsulas correspondente à dose de bicarbonato de sódio.

*Coletas Sanguíneas*

Para a análise do pH, bicarbonato e lactato serão colhidas amostras de 500 microlitros de sangue da veia antecubital. Durante as sessões experimentais, as amostras de sangue serão colhidas pré-suplementação, 90 minutos após a suplementação e imediatamente após o teste físico.

O lactato sanguíneo será analisado em um analisador automatizado (YSI 2300 – Yellow Springs – Ohio). As amostras serão armazenadas em tubos contendo solução de fluoreto de sódio a 2% para serem posteriormente analisadas eletroquimicamente no analisador automatizado de lactato. O pH, pressão parcial de O_2_ e CO_2_ sanguíneos serão avaliados por gasometria venosa em um analisador de gases sanguíneos. Para tanto será utilizado o RAPIDPOINT® 350 (Siemens, Alemanha). A concentração de bicarbonato será calculada segundo a equação de Andersen-Hasselbach.

*Avaliação da Capacidade Física*

A capacidade física será avaliada por meio do teste de capacidade cíclica a 110% da potência máxima (do inglês, Cycling Capacity Test - CCT_110%_) (Sale et al., 2011; Saunders et al., 2013). Este teste é realizado com uma carga fixa em 110% da potência máxima previamente determinada em um teste incremental até a exaustão. A posição do ergômetro, altura do banco e altura do selim serão gravadas durante os testes preliminares e mantidas ao longo das sessões experimentais. Após um aquecimento de 5 minutos, os participantes pedalarão na intensidade fixa até a exaustão voluntária ou que os participantes não consigam manter a cadência de 60 rpm. Serão avaliadas as variáveis trabalho total realizado (TT) e tempo até a exaustão (TTE).

*Análise Estatística*

Será realizada a *ANOVA-two way* (análise da variância com dois fatores) de medidas repetidas para detectar diferenças no pH, lactato e bicarbonato sanguíneos resultantes do tempo e tratamento. Separadamente, será realizado a *ANOVA-one way* (análise da variância com um fator) para detectar diferenças entre os tratamentos no trabalho total desempenhado no teste de CCT_110%_. Serão testadas 4 diferentes matrizes de co-variância para verificarmos qual o melhor modelo que se ajusta aos dados, de acordo com o critério de Schwarz Bayesian (menor valor BIC – “Bayesian Information Criterion”). No caso de um valor F significante, um teste de *post-hoc* de Tukey será utilizado para encontrar diferenças específicas. O nível de significância assumido será p<0.05.

**Riscos**

A única mensuração invasiva desse estudo são as coletas de pequenas amostras sanguíneas (500 µL) da veia antecubital de cada participante. Apesar do pequeno desconforto e dor dessa medida, nosso grupo possui experiência na mesma e dificilmente observa tais efeitos. Adicionalmente, apesar de serem raras, complicações cardíacas podem ocorrer durante testes máximos. Contudo, todos os testes máximos sempre serão executados com o acompanhamento de um médico cardiologista. Além disso, o teste físico proposto pode causar mal-estar devido à alta intensidade em que será executado. Entretanto, é importante salientar que todas os participantes passarão por familiarização e aquecimento adequando para os testes. Por fim, é possível que haja certo desconforto gastrointestinal com a suplementação proposta. Porém, com o intuito de atenuar tal efeito colateral, utilizaremos cápsulas gelatinosas.

**Beneficios**

Os participantes da pesquisa terão acesso gratuito à avaliação do seu condicionamento físico, por meio do teste de potência máxima e do teste físico que serão empregados. Além disso, todos os participantes poderão requisitar orientação nutricional a partir dos recordatórios alimentares que serão conduzidos no estudo.

**Orçamento Financeiro**

A equipe será composta por integrantes do LABNUTRI. Nosso grupo tem plena estrutura e condição financeira para a execução dos testes e das análises propostas nesse projeto, não sendo necessária a solicitação de recursos financeiros.

**Plano de Trabalho Cronograma**

- 2º semestre de 2013 (novembro e dezembro) – Submissão do projeto ao Comitê de Ética em Pesquisa;
- 1° semestre de 2014 (janeiro, fevereiro e março) - Processo de recrutamento e seleção de voluntários;
- 1º semestre de 2014 (abril, maio e junho) – Coleta de Dados;
- 2º semestre de 2015 (agosto e setembro) – Análise estatística dos resultados;
- 2º semestre de 2015 (outubro, novembro e dezembro) – Publicação dos resultados.

**Referencias Bibliograficas**

Allen DG, Lamb GD, Westerblad H. Impaired calcium release during fatigue. *Journal of Applied Physiology*, v. 104, n. 1, p. 296-305, 2008.

Artioli GG, Gualano B, Coelho DF, Benatti FB, Gailey AW, Lancha JR AH. Does sodium-bicarbonate ingestion improve simulated judo performance? *International Journal of Sport Nutrition and Exercise Metabolism*; v. 17, p. 206-217, 2007.

[Donaldson SK](http://www.ncbi.nlm.nih.gov/pubmed?term=%22Donaldson%20SK%22%5BAuthor%5D), [Hermansen L](http://www.ncbi.nlm.nih.gov/pubmed?term=%22Hermansen%20L%22%5BAuthor%5D), [Bolles L](http://www.ncbi.nlm.nih.gov/pubmed?term=%22Bolles%20L%22%5BAuthor%5D). Differential, direct effects of H+ on Ca2+ -activated force of skinned fibers from the soleus, cardiac and adductor magnus muscles of rabbits. *European Journal of Physiology*[*,*](http://www.ncbi.nlm.nih.gov/pubmed?term=Differential%20direct%20effects%20of%20H%2B%20and%20Ca2%2B%20-activated%20force%20of%20skinned%20fibres%20from%20the%20soleus%2C%20cardiac%2C%20adductor%20magnus%20muscle%20of%20rabbits) v. 376, n. 1, p. 55-65, 1978.

Fabiato A, Fabiato F. Effects of pH on the myofilaments and the sarcoplasmic reticulum of skinned cells from cardiac and skeletal muscles. *The Journal of Physiology*, v. 276, p. 233-255, 1978.

Harris RC, Edwards RH, Hultman E, Nordesjo LO, Nylind B, Sahlin K. [The time course of phosphorylcreatine resynthesis during recovery of the quadriceps muscle in man.](http://www.ncbi.nlm.nih.gov/pubmed/1034909) *European Journal of Physiology*, v. 367, n. 2, p. 137-142, 1976.

Horswill CA, Costill DL, Fink WJ, Flynn MG, Kirwan JP, Mitchell JB, Houmard JA. Influence of sodium bicarbonate on sprint performance: relationship to dosage. *Medicine and Science in Sports and Exercise*; v. 20, p. 556-569, 1988.

Katz A, Costill DL, King DS, Hargreaves M, Fink WJ. Maximal exercise tolerance after induced alkalosis. *International Journal of Sports Medicine*; v. 5, p. 107-110, 1984.

Linderman J, Kirk L, Musselman J, Dolinar B, Fahey TD. The effects of sodium bicarbonate and pyridoxine-alpha-ketoglutarate on short-term maximal exercise. *Journal of Sports Sciences*; v. 10, p. 243-253, 1992.

Mainwood GW, Worsley-Brown PA. The effect of extracellular pH and buffer concentration on the efflux of lactate from frog sartorius muscle. *The Journal of Physiology*, v. 250, n. 1, p. 1-22, 1975.

Mainwood GW, Cechetto D. The effect of bicarbonate concentration on fatigue and recovery in isolated rat diaphragm muscle. *Canadian Journal of Physiology and Pharmacology*, v. 58, n. 6, p. 624-632, 1980.

McNaughton L. Bicarbonate ingestion: effects of dosage on 60 s cycle ergometry. *Journal of Sports Sciences*; v. 10, p. 415-423, 1992.

Painelli VS, Roschel H, De Jesus F, Sale C, Harris RC, Solis MY, Benatti FB, Gualano B, Lancha Jr AH, Artioli GG. The Ergogenic Effect of Beta-Alanine Combined With Sodium Bicarbonate on Swimming Performance. *Applied Physiology, Nutrition and Metabolism*; v. 38, p. 525-532, 2013.

Price MJ, Simons C. The effect of sodium bicarbonate ingestion on high-intensity intermittent running and subsequent performance. *Journal of Strength and Conditioning Research*; v. 24, p. 1834–1842, 2010.

Requena B, Zabala M, Padial P, Feriche B. [Sodium bicarbonate and sodium citrate: ergogenic aids?](http://www.ncbi.nlm.nih.gov/pubmed/15705037) *Journal of Strength and Conditioning Research*, v. 19, n. 1, p. 213-224, 2005.

Sahlin K. Metabolic factors in fatigue. *Sports Medicine*, v. 13, n. 2, p. 99-107, 1992.

Sale C, Saunders B, Hudson S, Wise JA, Harris RC, Sunderland CD. Effect of β-alanine plus sodium bicarbonate on high-Intensity cycling capacity. *Medicine and Science in Sports and Exercise*; v. 43, p 1972-1978, 2011.

Saunders B, Sale C, Harris RC, Sunderland C. Reliability of a high-intensity cycling capacity test. *Journal of Science and Medicine in Sport*; v. 16, p. 286-289, 2013.

Saunders B, Sale C, Harris RC, Sunderland C. Sodium Bicarbonate and High-Intensity Cycling Capacity: Variability in Responses. *International Journal of Sports and Physiological Performance*. In press.

Sutton JR, Jones NL, Toews CJ. [Effect of PH on muscle glycolysis during exercise.](http://www.ncbi.nlm.nih.gov/pubmed/7261554) *Clinical Science (London, England: 1979)*, v. 61, n. 3, p. 331-338, 1981.
