# Supplementary material for: (In)Consistencies in Responses to Sodium Bicarbonate Supplementation: A Randomised, Repeated Measures, Counterbalanced and Double-Blind Study
Source: PLoS One. 2015 Nov 17;10(11):e0143086. doi: 10.1371/journal.pone.0143086 (PMC4648485; doi:10.1371/journal.pone.0143086)
Supplement: S2 Text — (DOCX) [file pone.0143086.s005.docx]

UNIVERSIDADE DE SÃO PAULO

ESCOLA DE EDUCAÇÃO FÍSICA E ESPORTE

LABORATÓRIO DE NUTRIÇÃO E METABOLISMO APLICADO À ATIVIDADE MOTORA

Efeitos da suplementação suplementação de bicarbonato de sódio sobre a capacidade física em múltiplos testes.

Principal investigator: Bryan Saunders

Supervisor: Prof. Dr. Bruno Gualano

DEPARTAMENTO DE BIODINÂMICA DO MOVIMENTO DO CORPO HUMANO

2013

**Summary**

Several studies have shown that muscle acidosis caused by H + ions accumulation in the muscle is a limiting factor for physical performance during high-intensity exercise. Thus, strategies in order to mitigate the fall of intramuscular pH have the potential to excel in sport. Among them, sodium bicarbonate supplementation has received special attention in recent years. Supplementation of this compound may increase the blood pH and bicarbonate levels, increasing extracellular buffering capacity, and hence, physical performance. Despite its proven metabolic effects, the efficacy of ergogenic supplementation sodium bicarbonate remains controversial. The inconsistencies in the results of research investigating the ergogenic efficacy of supplementation with sodium bicarbonate can be partially attributed to: administration of different doses of the supplement, exercise models that are not limited by intramuscular acidosis, individual variation in response to supplementation and gastrointestinal discomfort affecting some individuals. In accordance with these assumptions, our group recently found that when the participants who experienced gastrointestinal distress were removed from the analysis, sodium bicarbonate supplementation improved total work done at 110% maximal power output. In addition, it was found that supplementation of sodium bicarbonate was not effective in all individuals, including participants with positive blood responses to supplementation.

The objective of this study is to determine if there is a consistency in the ergogenic effect of sodium bicarbonate in individuals that improve physical capacity, and verify that the lack of response is also consistent among individuals whose physical capacity does not improve. For this, we recruited about 20 physically active men. This study design will have a randomized, placebo-controlled, double-blind cross-over, which the subjects are allocated to two different treatments: placebo and sodium bicarbonate (300mg / kg). For each of the treatments, subjects will undergo physical capacity tests to check changes in this parameter due to the treatments. Before each visit the diet of the volunteers will be standardised. Descriptive variables will be analysed (mean, median and standard deviation), and analysis of data quality (check if the distribution is normal and if the variance is homogeneous).

**Introduction**

Muscle fatigue is a phenomenon studied for decades and is characterized by skeletal muscle inability to maintain a certain tension or keep exercise at a given intensity (Sahlin, 1992). To this day the causes for its occurrence remain poorly understood. Nevertheless, evidence indicates particularly important roles of certain events in the onset of fatigue, such as the inhibition of enzymes that participate in energy transfer, the decrease in sensitivity to calcium ions (Ca^2+^) in the troponin site, decreased release or re-uptake of Ca^2+^ in the sarcoplasmic reticulum and depletion of energy substrates (Sahlin, 1992; Allen et al., 2008).

Nevertheless, with regard to high-intensity exercise, some causes have gained prominence in literature. Among them, the accumulation of certain metabolites within the muscle cell, in particular, H^+^ ions, leading to a decrease in muscle pH, i.e. an intramuscular acidosis. In this sense, studies have shown that H^+^ ions have the ability to compete with Ca^2+^ ions for the troponin binding site, hampering the ability of the contractile machinery to operate effectively (Donaldson et al, 1978;. Fabiato & Fabiato, 1978). Furthermore, the decrease in muscle pH caused by H^+^ ions can lead to inhibition of the resynthesis of phosphorylcreatine (Harris et al., 1976) and inhibition of key enzymes of the glycolytic pathway (Sutton et al., 1981), limiting the process of energy production for muscle contraction. Given this importance of pH control during high-intensity exercise, strategies that contribute to the maintenance of acid-base balance are potentially ergogenic.

In this sense, sodium bicarbonate supplementation has received special attention in the literature. Several studies have shown that sodium bicarbonate supplementation significantly increases the blood pH and bicarbonate concentrations in the blood (Requena et al., 2005). These changes increase the flow of H^+^ and lactate ions out of active muscle. This is due to increased activity of lactate cotransporter/H^+^ called monocarboxylate transporters, which become active as the intracellular/extracellular gradient of H^+^ ions increases (Mainwood & Worsley-Brown, 1975 ; Mainwood & Cechetto, 1980). Therefore, intramuscular pH decreases more slowly, with lower interference of acidosis on the contractile process and ATP production via glycolysis, and hence delaying the onset of fatigue.

Despite its proven metabolic effects, ergogenic efficacy of sodium bicarbonate supplementation remains controversial (Price & Simons, 2010). The inconsistencies in the results of research investigating the ergogenic efficacy of supplementation with sodium bicarbonate can be partially attributed to: administration of different doses of the supplement (Horswill et al, 1988), exercise models that are not limited by the intramuscular acidosis (Linderman et al., 1992), individual variation in response to supplementation (Price & Simons, 2010) and gastrointestinal discomfort affecting some individuals (McNaughton, 1992). In line with these assumptions, Saunders et al. (2014) found that when the participants who experienced gastrointestinal distress were removed from the analysis, sodium bicarbonate supplementation improved the total work done. In addition, the authors found that supplementation of sodium bicarbonate was not effective in all individuals, including participants with positive blood responses to supplementation.

Hypothesis - The performance and response in multiple blood tests are not consistent with the supplementation of sodium bicarbonate in the same individual.

Objectives:

General Objective: To investigate the effect of sodium bicarbonate on physical capacity during consecutive tests.

Specific Objective: To determine if there is consistency of the ergogenic effect of sodium bicarbonate in individuals that improve physical capacity, and verify that the lack of response is also consistent among individuals whose physical capacity does not improve.

Methodology

Sample selection

20 male subjects between 18-30 years will be selected to participate. After a detailed explanation of the research, risks and benefits involved, all participants must sign the consent form. To be included in this study, participants should be 1) healthy, with no cardiovascular disease or impairment of the locomotor system; 2) physically active, that is, physically active, however, without being engaged in any regular program of training and / or competition. They will be excluded from study participants who are making use or have made use of creatine and beta-alanine for the last 3 and 6 months respectively.

Location and Duration of Study

Blood tests will be conducted in LABNUTRI (Nutrition Laboratory and Metabolism Applied to Motor Activity) of EEFE-USP (School of Physical Education and Sports, University of São Paulo). The physical tests will be conducted in LADESP (Determinants Energy Laboratory of Sports Performance) of EEFE-USP.

The study will last approximately two semesters.

Experimental Design

The protocol will consist of nine visits to the laboratory, which will always be maintained at 19.0 ° C with a relative humidity of about 64%.

In order, the first visit will consist of a maximal power output test. The second visit will consist of a familiarization to the main physical capacity test. The subsequent experimental sessions are sessions where the volunteers are subjected to two different treatments, which are separated by washout periods of at least 48 hours: Sodium bicarbonate supplementation (300 milligrams per kilogram of body weight) and placebo supplementation (calcium carbonate, the same dosage of sodium bicarbonate).

The experimental sessions will be conducted in a double-blind, crossover countered, and placebo-controlled manner. During the experimental sessions, blood collections for the evaluation of pH are performed, blood lactate and bicarbonate before intake of supplements 90 minutes after ingestion thereof and immediately after the exercise test. All physical tests will be performed on the same equipment (Lode, Netherlands), where volunteers will be tested for time to exhaustion and total work done.

The experimental sessions will be conducted in the afternoon. In order to minimize the differences in the initial concentrations of muscle glycogen, our group will standardize the breakfast and lunch that precedes each of the experimental tests (75.6 ± 4.5 kJ / kg, containing 41 ± 1% carbohydrate, 19 ± 1% protein and 40 ± 1% fat). Participants will be asked to keep a food record of the last 24 hours before each test. They will also be instructed not to perform any physical activity the day before each test. They hold the standard breakfast at 09: 00hs in the morning, lunch at 12: 00hs and submit to the laboratory for physical testing and blood sampling at 14: 00hs afternoon. All testing and sampling will be conducted at the same time.

Supplementary Protocol

An acute supplementation of sodium bicarbonate and placebo will be adopted. The dose of sodium bicarbonate utilized is 300 milligrams per kilogram of body weight in order to try to mimic the doses used in previous studies that have detected an ergogenic effect of this nutritional strategy (Artioli et al, 2007;.. Painelli et al, 2013). The supplement is administered in gelatine capsules of the same size and color. The placebo used is calcium carbonate. To maintain the double blind design, it is administered in the same size and color of the sodium bicarbonate capsules. In addition, a placebo will be administered in a quantity corresponding to the dose of the sodium bicarbonate capsules.

Blood collections

For the analysis of pH, bicarbonate and lactate samples 500 microliters of blood are collected from the antecubital vein. During the experimental sessions, blood samples will be collected pre-supplementation, 90 minutes after supplementation and immediately after exercise test.

The blood lactate will be analyzed in an automated analyzer (YSI 2300 - Yellow Springs - Ohio). The samples are stored in tubes containing sodium fluoride solution to be 2% then electrochemically analyzed in the automated analyzer lactate. The pH, partial pressure of O2 and CO2 blood gases are evaluated intravenously in a blood gas analyzer. For that will be used RAPIDPOINT® 350 (Siemens, Germany). The bicarbonate concentration is calculated according to the equation of Andersen-Hasselbach.

Evaluation of Physical Capacity

Physical capacity will be assessed by the cycling capacity test at 110% of maximum power output (CCT^110%^; Sale et al, 2011; Saunders et al, 2013.). This test is performed with a fixed load at 110% of maximum power previously determined in an incremental test to exhaustion. The position of the ergometer, seat height and seat height will be recorded during the preliminary tests and maintained throughout the experimental sessions. After a warm-five minutes, participants will pedal at a fixed intensity to volitional exhaustion or participants can not keep a cadence of 60 rpm. The variables total work done (TWD) and time to exhaustion (TTE) will be recorded.

Statistical Analysis

Data will be analysed by two way ANOVA with repeated measures to detect differences in pH, lactate and blood bicarbonate resulting time and treatment. Separately, there will be a one way ANOVA (analysis of variance with one factor) to detect differences between treatments in total work performed in the CCT_110%_ test. 4 different matrices of covariance will be tested in order to verify what the best model that fits the data, according to the criterion of Schwarz Bayesian (BIC lower value - "Bayesian Information Criterion"). In case of a significant F value, Tukey post-hoc test will be used to find specific differences. The significance level will be P <0.05.

Risks

The only invasive measurement of this study are collections of small blood samples (500 uL) from the antecubital vein of each participant. In addition, although rare, cardiac complications may occur during maximum tests. However, all maximum tests are always performed with the accompaniment of a cardiologist. Furthermore, the proposed physical test may cause discomfort due to the high intensity that will be executed. However, it is important to note that all the participants will undergo familiarization of the test protocols. Finally, there may be some gastrointestinal discomfort with supplementation of sodium bicarbonate. However, in order to alleviate such side effects, we will use gelatin capsules.

Benefits

Participants will have free access to assess their fitness through the maximum power test and physical test to be employed. In addition, all participants can request nutritional counselling from dietary recalls that will be conducted in the study.

Budget Financial

The team will consist of LABNUTRI members. Our group has full structure and financial condition for the execution of tests and analyses proposed in this project, the request for funding is not necessary.

Work Plan Schedule

• 2nd half of 2013 (November and December) - Submission of the project to the Research Ethics Committee;

• 1st half of 2014 (January, February and March) - The process of recruitment and selection of volunteers;

• 1^st^ half of 2014 (April, May and June) - Data Collection;

• 2^nd^ half of 2014 (August and September) - Statistical analysis of results;

• 2^nd^ half of 2014 (October, November and December) - Publication of the results.
